# Patient and clinician experience of a serious illness conversation guide in oncology: A descriptive analysis

**DOI:** 10.1002/cam4.3102

**Published:** 2020-05-04

**Authors:** Joanna Paladino, Luca Koritsanszky, Lauren Nisotel, Bridget A. Neville, Kate Miller, Justin Sanders, Evan Benjamin, Erik Fromme, Susan Block, Rachelle Bernacki

**Affiliations:** ^1^ Ariadne Labs Brigham and Women's Hospital Harvard T.H. Chan School of Public Health Boston MA USA; ^2^ Harvard Medical School Boston MA USA; ^3^ Department of Medicine Brigham and Women's Hospital Boston MA USA; ^4^ University of Pennsylvania Philadelphia PA USA; ^5^ Division of General Internal Medicine and Primary Care Brigham and Women's Hospital Boston MA USA; ^6^ Department of Psychosocial Oncology and Palliative Care Dana‐Farber Cancer Institute Boston MA USA; ^7^ Department of Psychiatry Brigham and Women's Hospital Boston MA USA

**Keywords:** advanced cancer, advance care planning, clinician experience, goals of care communication, palliative care, patient experience, prognostic communication, serious illness communication

## Abstract

**Background/objective:**

Oncology guidelines recommend earlier communication with patients about prognosis and goals‐of‐care in serious illness. However, current evidence leaves gaps in our understanding of the experience of these conversations. This analysis evaluates the patient and clinician experience of a conversation using a Serious Illness Conversation Guide (SICG).

**Design/setting:**

Secondary analysis from a cluster‐randomized clinical trial in a northeastern cancer center.

**Participants:**

Physicians, advanced practice clinicians, and patients with advanced cancer who received the intervention.

**Intervention:**

SICG, clinician training, systems‐changes.

**Main outcomes and measures:**

The patient questionnaire assessed perceptions of the conversation and impact on anxiety, hopefulness, peacefulness, sense of control over medical decisions, closeness with their clinician, and behaviors. The clinician questionnaire assessed feasibility, acceptability, and impact on satisfaction in their role.

**Results:**

We enrolled 54 clinicians and 163 patients; 41 clinicians and 118 patients had a SICG discussion. Most patients described the conversation as worthwhile (79%) and reported no change or improvement in their sense of peacefulness, hopefulness, and anxiety (on average 79%); 56% reported feeling closer with their clinician. Qualitative patient data described positive behavior changes, including enhanced planning for future care and increased focus on personal priorities. Nearly 90% of clinicians agreed that the SICG facilitated timely, effective conversations, and 70% reported increased satisfaction in their role.

**Conclusion:**

Conversations using a SICG were feasible, acceptable, and were associated with positive experiences for both patients and clinicians in oncology in ways that align with national recommendations for serious illness communication. This trial is registered at ClinicalTrials.gov: NCT01786811 https://clinicaltrials.gov/ct2/show/NCT01786811.

## Graphical abstract

This descriptive analysis reports the patient and clinician experience of a structured conversation about patients' values, goals, prognosis, and care preferences in the outpatient setting in oncology. Conversations using the Serious Illness Conversation Guide were feasible, acceptable, and were associated with positive experiences for both patients with advanced cancer and oncology clinicians in ways that align with national recommendations for serious illness communication.

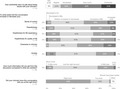

## 1 INTRODUCTION

Communication between clinicians and patients with serious illness about values and goals (“serious illness communication”) shapes the patient experience of care.^1^ Many interventions have been developed to improve serious illness communication; however, studies about their impact on patient experience reveal contradictory findings.^2^, ^3^, ^4^, ^5^, ^6^, ^7^, ^8^, ^9^, ^10^ For example: some studies demonstrate improvements in patient satisfaction, trust in their clinician, quality of communication, or well‐being,^3^, ^5^, ^6^, ^7^, ^8^, ^10^ while others have shown null results on these outcomes or worsening of patient distress.^2^, ^4^, ^6^, ^9^ Furthermore, among the most critical elements of any communication intervention are its acceptability, usability, and impact on clinicians, given that communication interventions often target clinician behavior change. While numerous studies describe clinicians’ perceptions of communication skills training or observed communication behaviors,^3^, ^9^, ^11^, ^12^ less is reported about the clinician experience of changing their own communication behaviors in practice.

Complicating the experience of these discussions are distress and anxiety. Clinicians worry about harming patients through disclosure of difficult information and express uncertainty about the appropriate time for such discussions.^13^, ^14^, ^15^ Patients want information about the anticipated course of their illness and at the same time feel anxious or ambivalent about receiving information about the future.^16^, ^17^, ^18^, ^19^, ^20^ In addition, less is known about the impact of communication on other key domains of patient experience: feeling known as a person,^21^, ^22^ heard and understood by their care team,^23^, ^24^ having a sense of control over their medical decisions,^21^, ^22^ or occurrence of shared decision‐making.^25^ Direct data from patients and clinicians about their experience of communication interventions would provide valuable insights, potentially filling in the gaps about how these interventions work.

To improve serious illness communication, we developed a communication quality improvement intervention, the Serious Illness Care Program, and tested it in a cluster‐randomized clinical trial in outpatient oncology. The foundation of this program is a Serious Illness Conversation Guide (SICG), a tool designed to support clinicians in having early, person‐centered conversations with patients about their values, goals, prognosis, and preferences.^7^, ^26^, ^27^, ^28^ In this manuscript, we report a descriptive analysis of the experience of this structured conversation for patients with advanced cancer and oncology clinicians in the intervention group.

## 2 STUDY DESIGN AND SETTING

This was a descriptive secondary aim to examine the patient and clinician experience of SICG‐led conversations in the context of a cluster‐randomized trial at the Dana‐Farber Cancer Institute (DFCI) (2012‐2016).^27^ Primary and secondary outcomes of this trial are reported elsewhere.^7^, ^26^ Clinicians were randomized in clusters to intervention or control. The study was approved by the DFCI Institutional Review Board.

Physicians and advanced practice clinicians from the DFCI were eligible. Eligible patients were at least 18 years old and identified by their oncology clinician with a “no” response to the surprise question: *Would you be surprised if this patient died in the next year?* ^29^ Clinicians and patients randomized to the intervention arm were included in this analysis. Oncology clinicians who specialize in melanoma and patients with metastatic melanoma were also enrolled in a third nonrandomized arm of the trial.^27^ These clinicians and patients received the same intervention components as the randomized intervention participants and were included in this analysis.

### 2.1 Intervention

The intervention included three components: (a) Clinical tools, including the SICG for clinicians, as well as materials to prepare patients and support their communication with family members; (b) Clinician training and coaching; (c) Systems‐changes, including an email reminder and electronic medical record documentation template. Clinicians received $150 gift cards for participation; patients did not receive remuneration. Table 1 lists the components of the SICG and rationale. Additional details about the study design and intervention are described elsewhere.^27^

**TABLE 1:** Elements of the serious illness conversation guide^a^, ^b^

Component	Description	Rationale	Patient‐tested Language
Setup	Clinicians **open** the discussion with gentle, relatable language to create safety	By normalizing the discussion and starting the conversation earlier when it is not necessary to make a decision, we create space for the patient to have time to process feelings, thoughts, and decisions. Asking permission helps to give the patient control.	Key tips for the setup^a^, ^b^ Thinking in advance Is this ok? (Asking permission) Benefits for the patient and family
Understanding	Clinicians **assess** the patient's awareness of their illness and illness course.	Clinician can titrate the discussion to the patient's understanding.	“What is **your understanding** now of where you are with your illness?”^a^, ^b^
Information Preferences	Clinicians **assess** the patient's preferences for information about the future, which varies from person to person.	Understanding the patient's desire for information ensures that the patient has control and avoids overwhelming the patient with intolerable information.	“**How much information** about what is likely to be ahead with your illness would you like from me?”^a^, ^b^
Prognosis	Clinicians **share** information about prognosis to the degree desired by the patient.	Sharing prognosis with patients is one of the hardest things we do as clinicians, and yet it is also a foundational element of shared decision‐making. Sharing patient‐centered information about the anticipated illness course, even if uncertain, enables patients to factor this information into their decision‐making and plans.	Key tips for sharing prognosis:^a^, ^b^ Use hope/worry or hope/prepare language to align with patients when sharing prognosis Allow silence and respond to emotions Avoid medical jargon
Goals	Clinicians **explore** the patient's goals with regard to their health, well‐being, and personal lives.	Asking about goals allows the patient to focus on things that are important to them and aids clinicians in tailoring a recommendation that addresses patients’ priorities and creates an individualized care plan.	“If your health situation worsens, what are your most important **goals**?”^a^, ^b^
Fears/ Worries	Clinicians **explore** the patient's fears and worries with regard to their illness and illness course.	Fears and worries about suffering, survival, and family well‐being contribute to patients’ distress. Creating space for patients to express worries can be therapeutic for patients	“What are your biggest **fears and worries** about the future with your health?”^a^, ^b^
Function	Clinicians **explore** the patient's views of critical abilities, as well as tolerable and intolerable states of function and quality of life	Patients have different views on functional impairment. An opportunity to express values and prior experiences that inform how patients’ define quality of life can provide key guidance on complex and difficult decisions.	“What **abilities** are so critical to your life that you can't imagine living without them?”^a^, ^b^
Trade‐offs	Clinicians **explore** trade‐offs that they are willing or not willing to make in order to achieve different outcomes	Exploring the patient's views on different types of care, such as hospitalizations, ICU stays, or invasive treatments and procedures, allows patients to reflect on potential tradeoffs and can promote informed decision‐making	“If you become sicker, **how much are you willing to go through** for the possibility of gaining more time?”^a^, ^b^
Family	Clinician **explore** the patient's wishes for the degree of family and caregiver involvement throughout the patient's illness course.	By exploring patients’ wishes for family involvement, clinicians and patients can partner to develop a plan for involving family members in important discussions, especially since family members may be involved in decisions.	“How much does your **family** know about your priorities and wishes?”^a^, ^b^
Recommendation	Clinicians **recommend** a plan for next steps based on the patient's priorities and the medical realities and options.	By guiding the clinician to incorporate a recommendation about next steps into the conversation, the goal is to provide support to the patient in deciding on next steps. Emotional support and guidance reduces anxiety.	“Based on what's important to you, I’d **recommend…**.. How does that sound?”^a^, ^b^

^a^ This table is adapted from the following article: American College of Physicians High Value Care Task Force. Communication about serious illness care goals: a review and synthesis of best practices. *JAMA Intern Med*. 2014;174(12):1994‐2003. https://doi.org/10.1001/jamainternmed.2014.5271

^b^ The Serious Illness Conversation Guide has been updated and is publicly available. The Serious Illness Conversation Guide. © 2015‐2017 Ariadne Labs: A Joint Center for Health Systems Innovation (www.ariadnelabs.org) at Brigham and Women's Hospital and the Harvard TH Chan School of Public Health, in collaboration with Dana‐Farber Cancer Institute. Licensed under the Creative Commons Attribution‐NonCommercial‐ShareAlike 4.0 International License, http://creativecommons.org/licenses/by‐nc‐sa/4.0/.

### 2.2 Patient involvement

Patients were involved in the design of the intervention. Patients were included in a National Advisory Panel that informed the intervention development. In addition, patients on the DFCI Patient and Family Advisory Council provided input to inform the intervention components and materials, including the Serious Illness Conversation Guide. Patients were also involved in the development of the survey that measured the main outcome for the trial (Life Priorities Survey). Further details are described elsewhere.^27^

### 2.3 Measures

Patient and clinician experience were measured with surveys administered to patients after their SICG conversation, and to clinicians after the first SICG conversation they had with a patient and again at the end of the study. With a few exceptions, all questions were in the form of 4‐ or 5‐point Likert scales with response sets appropriate to the question. During the trial, the surveys were revised: wording was clarified on some questions, Likert response options changed on some, and additional questions were added. We present results for the questions whose initial and revised forms are comparable, and we pooled data for the analysis. We label the questions/responses according to their revised form (the original and revised form of the questions are presented in the online Table S1).

#### 2.3.1 Baseline characteristics

Patients and clinicians in the intervention and melanoma arms completed baseline demographic surveys.

#### 2.3.2 Patient experience

Patients reported the impact of the conversation on their peacefulness, hopefulness about quality of life and life expectancy, sense of closeness with their clinician, and anxiety. Patients reported the appropriateness of the amount of information received and the timing of the discussion, as well as the extent to which the conversation was worthwhile. The questionnaire also contained a qualitative question: “What, if anything, have you done differently as a result of this discussion?” Patients responded in free text.

#### 2.3.3 Clinician experience

For feasibility, clinicians reported on the SICG’s ease of use, simplicity, and efficiency. Regarding acceptability, clinicians reported the extent to which: the SICG enabled them to evaluate patients’ prognostic understanding and titrate prognostic information to patient needs; was useful in helping them understand patients’ goals, fears, critical abilities, and preferences for aggressive treatments and family communication; was effective in helping them understand patient values and goals overall; they planned to use the SICG after the trial; the SICG enhanced the clinical care of the patient; and they learned something surprising about the patient. Clinicians were also asked whether or not they would want a SICG discussion if they were seriously ill (yes, no).

Further, clinicians reported their perceived impact of the SICG discussion on patient emotions, satisfaction with their role in patient care, and their anxiety in having these discussions.

We used self‐designed patient and clinician survey instruments derived from an extensive literature review; questions were reviewed for face validity by experts in palliative care, psychiatry, geriatrics, and survey methods.^28^ A secure web‐based application was used to collect and manage all study data.

### 2.4 Data analysis

#### 2.4.1 Baseline characteristics

We report descriptive statistics for patient and clinician characteristics. Adjusting for study cluster, we report mean and 95% confidence intervals (CI) for continuous variables and counts and frequencies for categorical variables.

#### 2.4.2 Patient and clinician experience

We performed descriptive analyses for patient and clinician experience survey questions and report counts and frequencies for each response category. For survey questions/responses whose wording changed during the study, we report the revised version.

#### 2.4.3 Qualitative analysis

We applied thematic content analysis to the patient responses to the open‐ended question using Excel.^30^, ^31^ Three authors (JP, LN, LK) independently read and coded a random sample of responses (20%) using open coding. They then met in person to develop a preliminary codebook with agreed upon categories and themes, into which codes were organized. A fourth author with qualitative research experience (JS) reviewed and helped refine the codebook by, for example, validating or proposing new organizational choices. JP, LN, and LK used this codebook to independently code remaining responses, iteratively refining the codebook as necessary. All responses were evaluated using the final codebook, and coders resolved discrepancies by consensus.

## 3 RESULTS

### 3.1 Patient and clinician characteristics

A total of 54 clinicians and 163 patients enrolled in intervention and melanoma arms. Of the 148 patients whose clinicians were triggered at least once, 118 patients (80%) had the SICG discussion. See Tables 2 and 3 for patient and clinician characteristics and Figure S1 for a CONSORT diagram.

**TABLE 2:** Patient characteristics

Characteristics	Patients who completed the questionnaire^a^ (n = 93)
	No. (%)
Age at baseline (years) (mean, 95% CI)	60 (58‐63)
Gender (%, n)	
Female	52 (56)
Male	41 (44)
Race	
White	85 (91)
Black	1 (1)
Other	6 (6)
Missing	1 (1)
Hispanic	
No	88 (95)
Yes	2 (2)
Missing	3 (3)
Married/partnered	
No	19 (20)
Yes	74 (80)
Income less than $75 k	
No	52 (56)
Yes	36 (39)
Missing	5 (5)
Disease center	
Breast oncology	22 (24)
GI, GU, Head and Neck, Neuro‐Onc, Sarcoma, Thoracic, Other	52 (56)
Heme, Lymphoma	4 (4)
Missing	15 (16)
Health Insurance	
Medicare	40 (43)
Medicaid/mass health	5 (5)
Private	46 (49)
Missing	2 (2)
Current health status	
Relatively healthy or not seriously ill	19 (20)
Relatively healthy but terminally ill	54 (58)
Seriously but not terminally ill	14 (15)
Seriously and terminally ill	6 (6)
Education high school or less	
No	74 (80)
Yes	19 (20)

^a^ Of the 93 patients, 46 completed an amended version of the same survey with 3 additional questions added. For information about the survey modifications and characteristics of this patient subgroup, please see Table S1.

**TABLE 3:** Clinician characteristics^a^

Characteristics	Clinicians who completed the questionnaire after 1st SICG conversation (n = 39)^a^
	No. (%)
Gender	
Female	25 (64)
Male	14 (36)
Clinician type	
MD	27 (69)
NP	10 (26)
PA	2 (5)
Disease center	
Breast oncology	9 (23)
GI, GU, head and neck, neuro‐onc, sarcoma, thoracic, other	20 (51)
Heme, Lymphoma	3 (8)
Missing	7 (18)
Years of practice in professional role (mean, 95% CI)	13 (9‐17)
Percentage of time spent on clinical duties (mean, 95% CI)	69 (59‐80)

^a^ 21 of the clinicians completed 4 additional questions at the end of the study. For additional information about the characteristics of this subgroup of clinicians, please see Table S1.

### 3.2 Patient experience

Of patients who had a SICG discussion with their clinician, 93 patients completed the survey (response rate 79%).

**FIGURE 1:**
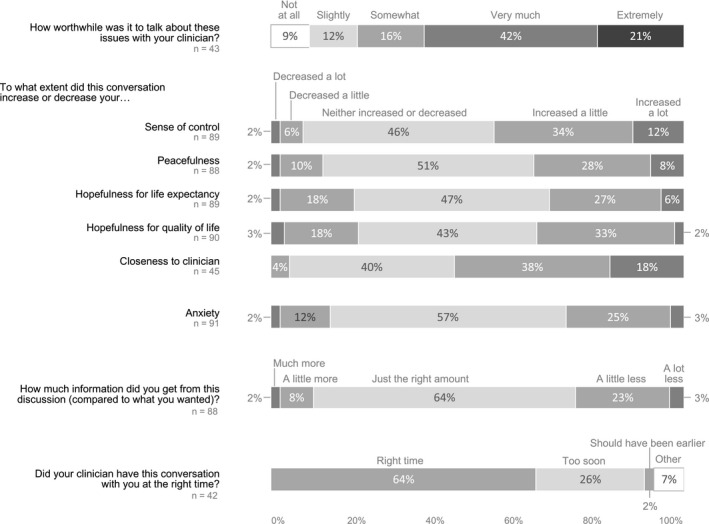
Patient experience of the SICG discussion

As shown in Figure 1, 79% of patients described the conversations as somewhat, very much, or extremely worthwhile. About 35% of patients reported increased peacefulness, hopefulness about their quality of life, and hopefulness about their life expectancy, while 56% reported increased closeness with their clinician and 46% reported increased sense of control over their medical decisions. Most of the remaining patients reported no change in these domains, with decreases reported by fewer than 20% of patients. A subset of patients reported decreased anxiety (14%) after the conversation, with most of the remaining patients reporting that anxiety was unchanged (57%) or increased (28%). Nearly two‐thirds of patients reported that they received the exact amount of information they wanted and that it was the right time to talk about these issues; 26% of patients reported that they received less information than they wanted.

### 3.3 Clinician experience

Of clinicians who had a SICG discussion (n = 41), 39 completed a survey after the initial conversation (response rate 95%).

**FIGURE 2:**
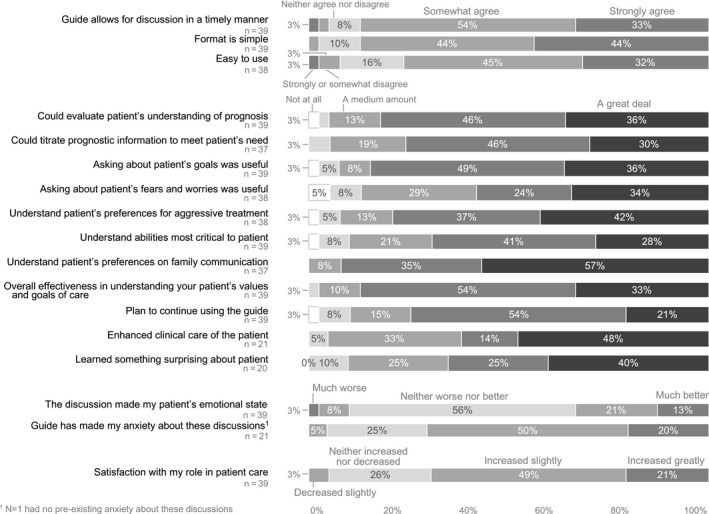
Clinician experience of the SICG discussion

As shown in Figure 2, clinicians found the SICG feasible, with about 90% agreeing that the SICG allowed discussion in a timely manner with a simple format; 77% of clinicians agreed that it was easy to use. Most clinicians reported that the SICG very much or a great deal: enabled them to evaluate patient understanding of prognosis (82%) and titrate prognosis according to patient preferences (76%); get useful information about goals (85%) and fears and worries (58%); understand the patient's critical abilities (69%), preferences for aggressive treatments (79%), preferences for family communication (92%) and values and goals overall (87%). Three‐quarters reported that they plan to use the SICG with patients after the trial (75%); the majority reported that the Guide enhanced their clinical care of the patient (62%) and they learned something surprising about their patient (65%). Nearly three‐fourths of clinicians perceived that their own anxiety in having these discussions improved (70%), with 90% perceiving that the discussion did not worsen the patient's emotional state. Of note, 70% reported that their satisfaction in their role increased and 86% reported that they would want to have a SICG discussion themselves if they were seriously ill.

### 3.4 Qualitative results: patient survey responses

As part of the survey, we asked patients to comment on what, if anything, they have done differently as a result of this conversation; 66% (n = 61) responded to this question and eleven patients of this group (18%) responded that they did not do anything differently. The remaining responses fell into six thematic categories, which we describe below with illustrative quotes. Patient responses occasionally fit into more than one category; most responses were brief, some only a few words.

#### 3.4.1 Enhanced planning of future medical care

In response to the conversation, patients considered and planned future medical decisions: “*…such as when I can no longer go [to the] bathroom by myself I would like hospice house care” (24*) or an increased awareness of *“items that I needed to communicate to my wife, ie hospice, ventilators” (41)*. Others described the conversation as helping them make treatment decisions, for example: *“whether chemo treatments might actually increase my longevity… [convincing] me to move ahead with chemo rather [than] quality of life care.” (37)*.

#### 3.4.2 Surfacing values and priorities

Patients described thinking *“more about my life and what's important” (9)*, becoming *“…more focused on goals I want to accomplish” (25)*, and considering *“… what my priorities are in terms of quality of life” (57)*. One patient described that the conversation “*…has made me able to recognize the importance of defining my values… which places a strong focus on the importance of nurturing…relationships with family and friends…” (39)*.

#### 3.4.3 Practical planning for their future

For many patients, the conversation prompted life planning: *“I made a list of things that I wanted to get accomplished in the near future. Some of it has to do with preparing my home and estate for the day when I can no longer do for myself. I feel at this point I am trying to get things done in order to make things easier for my family” (7)* Other examples are: *“…it made me realize that I need to take accessibility into account as a criterion” (10)*.

#### 3.4.4 Communication with family members

The conversation facilitated sharing of information and thoughts with family members: *“It gave me focus and I felt relieved after I spoke about some difficult stuff with them” (42); “Sharing this conversation later with my spouse and close family/friends allowed sharing my thoughts…and, in turn, hearing new ideas and supportive words” (49)*. The conversation also allowed patients to include or be more considerate of their families: *“Dr X’s question helped me reflect upon how I can be more sensitive to my [partner's] needs” (39)*; *“I also talked to my two grown children about how I’m doing. I don't want to keep them in the dark so nothing will be a shock.” (18)*.

#### 3.4.5 Positive changes in well‐being and relationship with clinician

Patients reported *“feeling less anxious about the future” (16),* feeling *“relieved”* after sharing the conversation with loved ones *(42)*, “*sleeping better”* (*60)*, and *“mostly, the conversation brought us closer* [with Dr]*.” (36)*.

#### 3.4.6 Conversation timing and context

For a small subset of patients, timing was perceived to be inappropriate: *“Given the current state of my treatment, this conversation was somewhat ill‐timed. At the moment… end of life issues are not primarily in my mind” (13)*. For some patients, the conversation felt *“too quick” (11),* or they felt they needed more time to “*think it through before I discuss and answer questions.” (31)*.

## 4 DISCUSSION

We found that a structured conversation about patients’ values, goals, prognosis, and care preferences using the SICG was feasible to implement and was associated with clinically meaningful and positive experiences for patients with advanced cancer and oncology clinicians.

These findings on clinician‐ and patient‐reported feasibility and acceptability of the SICG in the intervention group must be interpreted in the context of published results on the effectiveness of the whole intervention. Compared to control, the intervention demonstrated no effect on goal‐concordant care or peacefulness at the end of life but did demonstrate significant improvements in the prevalence, timing, and comprehensiveness of documented serious illness conversations as well as significant reductions in patients’ anxiety and depression symptoms.^7^, ^26^

Patients with serious illness often experience fear and loss of control.^16^, ^32^ Planning and preparing for the future can enhance control, and nearly half of the patients in this study reported increased control over their medical decisions. This intervention, which was designed to initiate at least one discussion, led patients toward specific actions: concrete planning for future care, life planning and prioritization, and enhanced communication with family members. All of these are known to be important to patients with serious illness.^18^, ^33^, ^34^ We postulate that the structure of the SICG, which focuses on prognostic disclosure, tradeoffs, and acceptable quality of life, and encourages reflection on goals, priorities, and family communication, may help patients feel more equipped to face subsequent health challenges and empowered to set and achieve goals and make decisions about their care.^34^, ^35^ Indeed, this may partly explain why nearly 80% of the patients found these conversations to be worthwhile.

Our findings demonstrate that for almost 75% of the patients, the SICG enabled a conversation with their clinician that did not cause increased distress. While the main contribution to this finding was that about half of patients did not report any change in elements of their experience after the conversation, it is also important to note that just over one‐third of patients reported that the structured conversation increased their sense of peacefulness and hopefulness and nearly 15% reported that their anxiety decreased, suggesting potential improvements in patient coping for some patients.^17^, ^36^, ^37^ A subset of patients, however, reported increased anxiety (mostly slight increases), which was not unexpected and hopefully transient. Reassuringly, patients in the intervention arm reported significantly lower rates of moderate‐severe anxiety and depression symptoms than control patients weeks after the conversation.^7^ We postulate that for most patients, the person‐centered focus of the SICG that emphasizes what is important to patients may protect against the worsening psychological distress highlighted by other studies.^2^, ^4^ In addition, a question about information preferences may prevent patients from receiving unwanted information.^19^ Nearly two‐thirds of patients reported that they received the right amount of information in this discussion, and one‐quarter wanted more information. While most patients reported that discussions happened at the right time, a small subset felt that the timing was too early or preparation was inadequate. Communication interventions may benefit from assessing patients’ readiness to discuss these issues or more actively preparing patients for the discussion in advance.^38^

Numerous studies document the barriers to having these conversations in oncology, including time constraints and concerns about patient discomfort.^13^, ^14^, ^15^ Most oncology clinicians in this trial found the SICG simple to use, efficient to build into practice, and useful in conducting the clinically meaningful activities mentioned above, including sharing prognosis according to patient needs and understanding patients’ values, goals, and preferences. This is supported by the clinician behavior changes that resulted from this intervention, including high utilization of the SICG and more, earlier, and better conversations.^26^ The fact that most clinicians expressed intention to continue using the SICG, described enhanced clinical care of their patients, and would want the tool to be used if they themselves were seriously ill, suggest a high degree of acceptance, which is a crucial component of behavior change interventions.^39^, ^40^, ^41^

These findings have potential implications for clinical practice. First, the SICG appears to be an effective tool for serious illness communication in oncology, which is necessary for shared decision‐making and thus a key element of high‐quality oncology care.^42^, ^43^, ^44^ Most patients had a sense that they received the right amount of information and most clinicians perceived that the SICG enabled them to effectively understand patients’ goals. Second, a clinician‐accepted tool that drives concrete behavior changes with safe, relatable language may help to overcome common obstacles to early communication in practice. Third, communication interventions that alleviate clinician anxiety and enhance satisfaction in their role may improve important clinician outcomes, including burnout. Moral distress, which is highly prevalent among those who care for people with serious illness, is increasingly recognized as a root cause of burnout.^45^, ^46^, ^47^, ^48^ Finally, these conversations have the potential to strengthen the patient‐clinician relationship (56% of patients reported enhanced closeness with their clinician), which may prompt further open discussions about goals and values and contribute to better patient care.

### 4.1 Limitations

The findings from this analysis must be interpreted within the context of its limitations. Patient and clinician surveys were not validated and may miss key elements of the experience of the SICG discussion. This analysis includes a small population of oncology clinicians and patients (mostly Caucasian) at a tertiary cancer center, which may differ from other populations. We changed the wording of some survey items, which may have biased responses in ways for which we have not accounted. We did not have a control group for this analysis, and we do not know if control patients would have reported something similar from usual care.

## 5 CONCLUSION

The SICG was feasible, acceptable, and was associated with positive experiences for both patients and clinicians in oncology in the intervention group in ways that align with national recommendations for serious illness communication. More research is needed to understand the effectiveness of serious illness communication interventions on improving the quality of the patient‐clinician relationship and on validated measures of patient and clinician experience.

## CONFLICT OF INTEREST

Dr Block receives compensation as Palliative Care Editor from Up to Date.

## AUTHOR CONTRIBUTIONS

Joanna Paladino, MD: Conceptualization, data curation, formal analysis, investigation, methodology, supervision, validation, writing original draft, writing—review, and editing. Luca Koritsanszky, RN, MPH: Data curation, formal analysis, methodology, project administration, writing—original draft, writing—review, and editing. Lauren Nisotel, BS: Data curation, formal analysis, project administration, writing—original draft, writing—review and editing. Bridget A. Neville, MPH: Conceptualization, data curation, formal analysis, methodology, software, validation, writing‐ original draft, writing‐ review and editing. Kate Miller, PhD: Conceptualization, formal analysis, methodology, software, validation, visualization, writing‐ original draft, writing‐ review and editing. Justin Sanders, MD, MSc: Conceptualization, formal analysis, methodology, writing‐ review and editing. Evan Benjamin, MS MD: Methodology, resources, supervision, writing‐ review and editing. Susan Block, MD: Conceptualization, methodology, supervision, writing—review and editing. Rachelle Bernacki, MD,MS: Conceptualization, funding acquisition, investigation, methodology, resources, supervision, writing—review and editing.

## Supporting information

Fig S1

Table S1

## Data Availability

The data that support the findings of this study are available on request from the corresponding author. The data are not publicly available due to privacy or ethical restrictions.
