# Supplementary figures and images for: Patient and clinician experience of a serious illness conversation guide in oncology: A descriptive analysis
Source: Cancer Med. 2020 May 4;9(13):4550–60. doi: 10.1002/cam4.3102 (PMC7333843; doi:10.1002/cam4.3102)

# Supplemental Figure 1: CONSORT Diagram

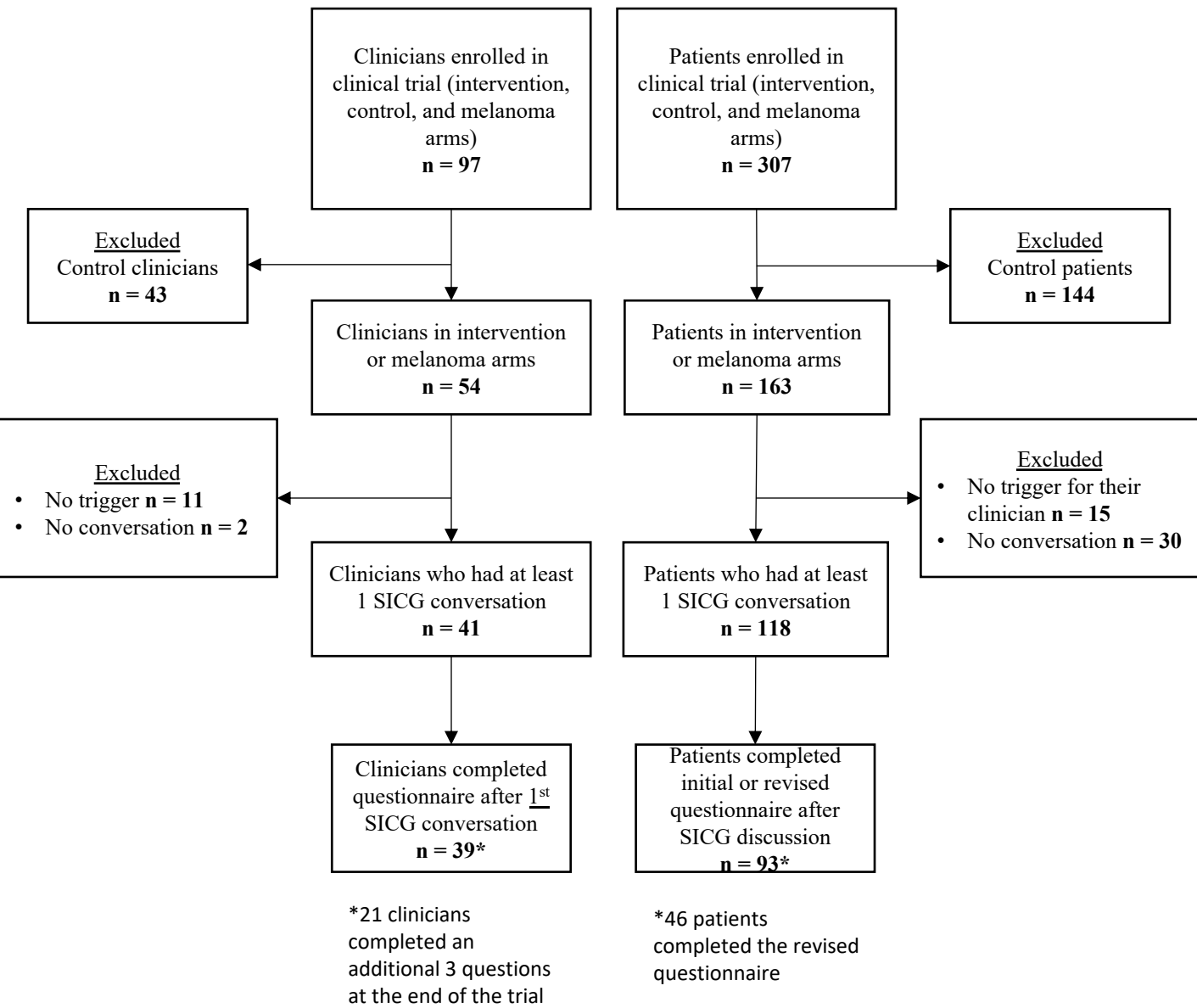

Supplement: Supplementary file 1 — Fig S1 [file CAM4-9-4550-s001.pdf]
