# Supplementary material for: Patient and clinician experience of a serious illness conversation guide in oncology: A descriptive analysis
Source: Cancer Med. 2020 May 4;9(13):4550–60. doi: 10.1002/cam4.3102 (PMC7333843; doi:10.1002/cam4.3102)
Supplement: Supplementary file 2 — Table S1 [file CAM4-9-4550-s002.docx]

**Supplemental Table 1**

**Patient questionnaire modifications^*^**

^*^The questionnaire was revised during the trial. Each patient completed only one questionnaire (either the initial form or the revised form). There were two types of revisions: 1) We added 3 new questions; these questions were only answered by patients who completed the revised form (n=46); 2) For a subset of questions that were included on both the initial and revised questionnaires, we changed the wording of the question or the response option. An expert psychometrician and research methodologist (KM) on the study team, in collaboration with the clinical faculty researchers (JP and RB), determined if the questions remained comparable from a scientific and clinical standpoint. The data were pooled across the initial and revised questionnaires. We labeled the questions/responses according to their revised form in the results section of the manuscript. All decisions about the survey items were made a priori.

| *Initial questionnaire* | | *Revised questionnaire* | | *What modification occurred* | *How was the question analyzed* |
| --- | --- | --- | --- | --- | --- |
| *Question* | *Response options* | Question | Response options |  |  |
| *To what extent did this conversation increase or decrease your anxiety about your illness?* | *1=greatly decreased 2=somewhat decreased 3=neither increased or decreased 4=somewhat increased 5=greatly increased* | Same | 1=decreased a lot 2=decreased a little 3=neither increased nor decreased 4=increased a little 5=increased a lot 6=Not applicable | Response options changed | Pooled |
| *To what extent did this conversation increase or decrease your sense of control over your medical decisions?* | *1=greatly decreased 2=somewhat decreased 3=neither increased or decreased 4=somewhat increased 5=greatly increased* | Same | 1=decreased a lot 2=decreased a little 3=neither increased nor decreased 4=increased a little 5=increased a lot 6=Not applicable | Response options changed | Pooled |
| *To what extent did this conversation increase or decrease your sense of peacefulness?* | *1=greatly decreased 2=somewhat decreased 3=neither increased or decreased 4=somewhat increased 5=greatly increased* | Same | 1=decreased a lot 2=decreased a little 3=neither increased nor decreased 4=increased a little 5=increased a lot 6=Not applicable | Response options changed | Pooled |
| *To what extent did this conversation increase or decrease your hopefulness about your prognosis?* | *1=greatly decreased 2=somewhat decreased 3=neither increased or decreased 4=somewhat increased 5=greatly increased* | To what extent did this conversation increase or decrease your hopefulness about your life expectancy? | 1=decreased a lot 2=decreased a little 3=neither increased nor decreased 4=increased a little 5=increased a lot 6=Not applicable | Response options and question changed | Pooled, results reported using the revised wording |
| *To what extent did this conversation increase or decrease your hopefulness about your quality of life in the future?* | *1=greatly decreased 2=somewhat decreased 3=neither increased or decreased 4=somewhat increased 5=greatly increased* | To what extent did this conversation increase or decrease your hopefulness about your quality of life? | 1=decreased a lot 2=decreased a little 3=neither increased nor decreased 4=increased a little 5=increased a lot 6=Not applicable | Response options and question changed | Pooled, results reported using the revised wording |
| *n/a* | *n/a* | To what extent did this conversation increase or decrease the closeness you have with your clinician? | 1=decreased a lot 2=decreased a little 3=neither increased nor decreased 4=increased a little 5=increased a lot 6=Not applicable | N/A | Revised only |
| *How much information did you get from this discussion?* | *1=I received much more information than I wanted 2=I received a little more information than I wanted 3=I received the exact amount of information I wanted 4=I received a little less information than I wanted 5=I received a lot less information than I wanted* | Same | Same | Item unchanged | Pooled |
| *n/a* | *n/a* | Did you feel that your clinician had this conversation with you at the right time? | 1=It was the right time to talk about this 2=It was too soon to talk about this, because it is too early in my relationship with my doctor 3=It was too soon to talk about this, because I am too health at this time 4=I wish my doctor had brought this up earlier 5=Other | N/A | Revised only |
| *n/a* | *n/a* | Overall, how worthwhile was it to talk about these issues with your clinician? | 1=not at all 2=slightly 3=somewhat 4=very much 5=extremely 6=have not had conversation | N/A | Revised only |
| *What, if anything, have you done differently as a result of this conversation?* | *open text* | Same | Same | Item unchanged | Pooled |

**Clinician questionnaire modifications^*^**

^*^Clinician questionnaires were sent after the 1^st^ SICG discussion and at the end of the trial; 39/41 clinicians completed the questionnaire after the 1^st^ SICG discussion and 21/41 clinicians completed the questionnaire at the end of the trial. A priori, we decided to report results from the questionnaire after the 1^st^ SICG discussion, given the higher ‘n’ for analysis. Since three questions were added to the questionnaire at the end of the trial, we decided a priori to include the results of those questions only from the ‘end of trial’ survey. We felt compelled to include these questions, even with the different timepoint and different ‘n,’ because of the exploratory nature of this analysis and their valuable contribution to the literature to inform future research studies.

| After 1^st^ conversation | | *End of study* | | *How did we analyze the question* |
| --- | --- | --- | --- | --- |
| Question | Response options | *Question* | *Response options* |  |
| I think that the SICG-guided discussion allows for end-of-life issues in a timely manner. | 1=strongly disagree 2=somewhat disagree 3=neither agree nor disagree 4=somewhat agree 5=strongly agree | *Same* | *Same* | *Survey after 1^st^ conversation only* |
| I think that the SICG-guided discussion format is simple | 1=strongly disagree 2=somewhat disagree 3=neither agree nor disagree 4=somewhat agree 5=strongly agree | *Same* | *Same* | *Survey after 1^st^ conversation only* |
| I think that the SICG-guided discussion is easy to use | 1=strongly disagree 2=somewhat disagree 3=neither agree nor disagree 4=somewhat agree 5=strongly agree | *Same* | *Same* | *Survey after 1^st^ conversation only* |
| To what extent were you able to evaluate your patient’s understanding of prognosis? | 1=Not at all  2  3=A medium amount  4  5=A great deal | *Same* | *Same* | *Survey after 1^st^ conversation only* |
| To what extent did asking about the patient’s information preferences allow you to titrate your delivery of prognostic information to meet the patient’s needs? | 1=Not at all  2  3=A medium amount  4  5=A great deal | *Same* | *Same* | *Survey after 1^st^ conversation only* |
| To what extent did you understand your patient's preferences regarding sharing of information with family member(s)? | 1=Not at all  2  3=A medium amount  4  5=A great deal | *Same* | *Same* | *Survey after 1^st^ conversation only* |
| To what extent did you gain useful information from asking about the patient’s goals? | 1=Not at all  2  3=A medium amount  4  5=A great deal | *Same* | *Same* | *Survey after 1^st^ conversation only* |
| To what extent did you gain useful information form asking about the patient’s fears and worries? | 1=Not at all  2  3=A medium amount  4  5=A great deal | *Same* | *Same* | *Survey after 1^st^ conversation only* |
| To what extent do you understand your patient’s preferences regarding undergoing aggressive treatments? | 1=Not at all  2  3=A medium amount  4  5=A great deal | *Same* | *Same* | *Survey after 1^st^ conversation only* |
| To what extent do you understand the abilities that are most critical to your patient? | 1=Not at all  2  3=A medium amount  4  5=A great deal | *Same* | *Same* | *Survey after 1^st^ conversation only* |
| Overall, how effective was this discussion in understanding your patient’s values and goals about end-of-life? | 1=Not at all  2  3=A medium amount  4  5=A great deal | *Same* | *Same* | *Survey after 1^st^ conversation only* |
| After the study is over, to what extent do you plan to continue to use the SICG format for discussing these issues with patients? | 1=Not at all  2  3=A medium amount  4  5=A great deal | *Same* | *Same* | *Survey after 1^st^ conversation only* |
| *n/a* | *n/a* | The Serious Illness Conversation Guide provided understanding that enhanced my clinical care of my patient. | 1=Not at all  2  3=A medium amount  4  5=A great deal | *Survey at the end of the trial only* |
| *n/a* | *n/a* | The Serious Illness Conversation Guide taught me something about my patient that surprised me | 1=Not at all  2  3=A medium amount  4  5=A great deal | *Survey at the end of the trial only* |
| I think the discussion of end-of-life issues made my patient’s emotional state: | 1=Much worse  2  3=Neither worse nor better  4  5=Much better | *Same* | *Same* |  |
| *n/a* | *n/a* | The Serious Illness Conversation Guide has made my anxiety about having these discussions: | 1=Much worse  2=Slightly worse  3=Neither worse nor better  4=Slightly better  5=Much better  6=I do not have anxiety about these discussions | *Survey at the end of the trial only* |
| Overall, how much did your discussion of these issues with your patient increase or decrease your satisfaction with your role in your patient’s care? | 1=Greatly decreased  2=Slightly decreased  3=Neither decreased or increased  4=Slightly increased  5=Greatly increased | *Same* | *Same* | *Survey after 1^st^ conversation only* |
| If you had a serious, life-threatening illness, would you want your clinician to have a values and goals discussion with you using this SICG? | 1=Yes  2=No | *Same* | *Same* | *Survey after 1^st^ conversation only* |

**Codebook for qualitative analysis of patient experience survey: patient responses to the open-ended question – What, if anything, did you do differently as a result of this conversation?**

| **Code Name** | **Definition** | **Example** |
| --- | --- | --- |
| Enhanced planning for future care | Stating preferences for care at the end of life or in preparation for changes in illness | *Made a complete list of all my last wishes, such as when I can no longer go bathroom by myself I would like hospice house care....(24)*  *I revised a document I have been working on entitled 'My Wishes for the End of My Life.' This conversation helped me to clarify my thoughts. (17)* |
| Increased focus on personal values and priorities | Focus on achieving a life goal or focus on personal values/priorities | *...has made me able to recognize the importance of defining my values... which places a strong focus on the importance of nurturing...relationships with family and friends... (39)*  *Determined my plans for the future on a 12 month basis, i.e., vacations, etc. (33)* |
| Increased attention to practical planning | Activities related to patients’ life planning such as planning a will or funeral | *..Some of it has to do with preparing my home and estate for the day when I can no longer do for myself. I feel at this point I am trying to get things done in order to make things easier for my family. (7)*  *Started my own list of things that I want taken care of while I still have the chance. Visited and chose a funeral director who I like and trust. Will be purchasing a burial plot soon. Have accomplished things on my list but still continue to add to it. (4)* |
| Enhanced communication with family members | More or better communication with family members about prognosis and/or illness | *I also talked to my two grown children about how I’m doing. I don’t want to keep them in the dark so nothing will be a shock. (18)*  *I used the guide she gave me to talk with my children (ages 21 and 24) about my illness. It gave me a focus and I felt relieved after I spoke about some difficult stuff with them. I was surprised about how much they understood and how strong they have both been. (42)* |
| Improved wellbeing | Feeling better in some way | *Sleeping better (60)*  *I am doing the same stuff as before, just feeling less anxious about the future (hope for the best, prepare for the worst). I live my life with a positive attitude. (16).* |
| Enhanced therapeutic relationship with clinician | Reports of feeling closer to (or more connected with) clinician | *Mostly, the conversation brought us closer. (36)*  *Immediately I felt gratitude for having a physician who could so aptly welcome me into conversation about this critical part of my experience and my medical care...I left with no sure answers except that [doctor] is an authentic part of the circle that is lifting me up and through this medical adventure. (49)* |
| Conversation too early or ill-timed | Expression that the conversation occurred abruptly or too early in the illness course | *Given the current state of my treatment, this conversation was somewhat ill-timed. At the moment ... end of life issues are not primarily in my mind (13)*  *I felt that having this conversation at this time especially when I was being so hopeful of getting better was inappropriate. (45)* |
| Nothing or no change | Didn’t do anything differently | *Nothing (51)*  *Nothing yet (54)* |

**Patient Characteristics of Subgroup**

| **Characteristics** | **Patients who completed the amended survey (n=46)** |
| --- | --- |
|  | **No. (%)** |
| Age at Baseline (years) (mean, 95% CI) | 62 (58-65) |
| Gender (%, n) |  |
| Female | 25 (54) |
| Male | 21 (46) |
| Race |  |
| White | 45 (98) |
| Black | 1 (2) |
| Other | 0 (0) |
| Missing | 0 (0) |
| Hispanic |  |
| No | 45 (98) |
| Yes | 0 (0) |
| Missing | 1 (2) |
| Married/Partnered |  |
| No | 10 (22) |
| Yes | 36 (78) |
| Income less than $75k |  |
| No | 28 (61) |
| Yes | 14 (30) |
| Missing | 4 (9) |
| Disease Center |  |
| Breast Oncology | 13 (28) |
| GI, GU, Head & Neck, Neuro-Onc, Sarcoma, Thoracic, Other | 30 (65) |
| Heme, Lymphoma | 3 (7) |
| Missing | 0 (0) |
| Health Insurance |  |
| Medicare | 19 (41) |
| Medicaid/Mass Health | 4 (9) |
| Private | 21 (46) |
| Missing | 2 (4) |
| Current Health Status |  |
| Relatively healthy or not  seriously ill | 7 (15) |
| Relatively healthy but terminally ill | 33 (72) |
| Seriously but not terminally ill | 4 (9) |
| Seriously and terminally ill | 2 (4) |
| Education high school or less |  |
| No | 37 (80) |
| Yes | 9 (20) |

**Clinician characteristics of subgroup**

| **Characteristics** | **Clinicians who completed additional questions at the end of the trial**  **(n=21)** |
| --- | --- |
|  | **No. (%)** |
| Gender |  |
| Female | 15 (71) |
| Male | 6 (29) |
| Clinician Type |  |
| MD | 14 (67) |
| NP | 6 (29) |
| PA | 1 (5) |
| Disease Center |  |
| Breast Oncology | 6 (29) |
| GI, GU, Head & Neck, Neuro-Onc, Sarcoma, Thoracic, Other | 13 (62) |
| Heme, Lymphoma | 0 (0) |
| Missing | 2 (10) |
| Years of practice in professional role (mean, 95% CI) | 14 (8-19) |
| Percentage of time spent on clinical duties (mean, 95% CI) | 77 (65-89) |
